# Transoral laser microsurgery for T1 glottic cancer with anterior commissure: Identifying clinical and radiological variables that predict oncological outcome

**DOI:** 10.1007/s00405-024-08513-3

**Published:** 2024-02-29

**Authors:** Caglar Eker, Ozgur Surmelioglu, Muhammed Dagkiran, Omer Kaya, Ilda Tanrisever, Burak Arpaci, Bedir Kaya, Sevinc Puren Yucel Karakaya, Elvan Onan

**Affiliations:** 1 Faculty of Medicine, Department of Otolaryngology and Head and Neck Surgery, Cukurova University, Campus of Balcali, Saricam, 01330 Adana, Turkey; 2 Faculty of Medicine, Department of Radiology, Cukurova University, Adana, Turkey; 3 Faculty of Medicine, Department of Biostatistics, Cukurova University, Adana, Turkey

**Keywords:** Transoral laser microsurgery, Anterior commissure, Early stage glottic cancer, Thyroid cartilage angle

## Abstract

**Purpose:**

The involvement of the anterior commissure (AC) is regarded to be a risk factor for poor results after transoral laser microsurgery (TLM) for early glottic cancer. The objective of this study was to determine how AC-related clinical and radiological factors affected oncological outcomes in a cohort of patients with T1 stage early glottic carcinoma involving the anterior commissure who were treated with TLM with negative surgical margins.

**Methods:**

Retrospective analysis was performed on clinical, radiological, and follow-up data of patients consecutively treated with TLM at a tertiary academic center between November 2011 and August 2021 for T1 glottic squamous cell carcinoma involving the anterior commissure. Recurrence-free survival (RFS), local control with laser alone (LCL), laryngeal preservation (LP), and overall survival (OS) rates (Kaplan–Meier) were the primary outcome metrics.

**Results:**

In our series, 5-year OS probability was 75.1%, RFS was 64.8%, LCL was 73.8%, and LP was 83.4%. OS and RFS were higher in patients with early stages of AC pattern than in patients with advanced stage (*p = *0.004, *p = *0.034, respectively). Vertical extension ratio was found to be associated with OS and RFS (*p = *0.023, *p = *0.001, respectively), and thyroid cartilage interlaminar angle with LCL by multiple Cox regression analysis (*p = *0.041).

**Conclusion:**

TLM remains a valuable treatment option for AC involvement. AC3 type involvement and elevated vertical extension ratio were associated with negative prognosis. There have been signs that thyroid cartilage with a narrow angle increases recurrence. Alternative modalities should be kept in mind in the treatment decision of these cases.

## Introduction

Transoral laser microsurgery (TLM) has been widely used in the treatment of early stage glottic squamous cell carcinoma (SCC) for the past few decades. Today, local control and laryngectomy-free survival rates after transoral organ preservation surgery using carbon dioxide (CO_2_) laser in early stage glottic cancer are quite satisfactory. In the management of early glottic cancer, the balance of oncological success and minimized treatment morbidity is of great importance [1].

Anterior commissure (AC) involvement in early glottic cancer was often suspected as a risk factor for recurrence after primary treatment with TLM [2]. Although there are those who advocate that Broyles ligament is a barrier to tumor invasion, the generally accepted view is that AC involvement is a poor prognostic factor [3]. On the other hand, it is important to distinguish between tumors located in the AC in the horizontal plane and tumors extending from the AC in the vertical plane to the supraglottis and/or subglottis in terms of predicting the prognosis. Complete exposure, appropriate endoscopic and radiological evaluation, and advanced surgical skills are imperative when dealing with tumors in this anatomical location. The unexpected difficulty in visualizing these lesions involving the anterior commissure can lead to incomplete surgical resection.

Computed tomography (CT) has long been recognized as an invaluable diagnostic procedure in patients with laryngeal cancer [4]. It is especially valuable in detecting cartilage invasion, supraglottic extension towards the pre-epiglottic area, subglottic extension towards the cricothyroid membrane, but has some limitations in early glottic cancer.

In the present study, we examined a cohort of T1 stage AC-involving glottic carcinomas with TLM-treated negative surgical margins. The objective of our study was to investigate the impact of clinical and radiological variables associated with the AC on the prognosis of the disease.

## Materials and methods

### Ethical consideration

This study was performed in line with the principles of the Declaration of Helsinki. Approval was granted by the Non-Invasive Clinical Research Ethics Committee of Cukurova University Faculty of Medicine.

### Study design

The records of patients treated by TLM for early stage glottic SCC involving AC at a tertiary university hospital from November 2011 to August 2021 were reviewed. The cases had not previously received any treatment for this reason. Preoperative diagnostic evaluation included panendoscopy with 0° and 70° rigid telescopes and cervical palpation. Contrast enhanced CT evaluation was performed to exclude spread to the paraglottic and pre-epiglottic space and cartilage invasion. In cases of massive trans-commissural vertical spread in AC, radiological evidence of erosion of the inner cortex of the thyroid cartilage, and involvement of the paraglottic space, patients were referred to other treatment options. Patients who could not provide adequate laryngeal exposure were also not considered suitable for TLM. Patients with concurrent malignancies, those who needed to switch to open surgery in the same session, and patients whose pre-operative radiology and follow-up data were not available were excluded from the study.

All patients underwent direct laryngoscopy under general anesthesia for detailed examination and histopathological diagnosis before laser surgery. The localization and spread pattern of the tumor were examined in detail, and biopsies were taken from different parts of the tumor, one of which was the AC, for histopathological diagnosis. The presence of tumor in AC of all patients was histopathologically confirmed. Those with T2 stage according to endoscopic examination, radiology and histopathology were excluded from the study. Resection margins were considered positive if tumor tissue extended to the border of the specimen or was < 1 mm from the nearest free margin. Patients with positive surgical margins underwent surgical margin enlargement or wait and see. These patients were excluded from the study.

### Surgical technique

All procedures were performed under general anesthesia. Suspension laryngoscopy was performed and the lesion was examined under the microscope. Type Va or VI cordectomy was performed according to the European Laryngology Association classification [5]. Cordectomies were performed using Sharplan 40C surgical CO_2_ laser system coupled to an AcuBlade micromanipulator (Lumenis, Israel) and a M525 F40 microscope (Leica, Germany). Resections were performed using the en bloc technique when the volume of the tumor allowed, while larger tumors were removed using the piecemeal technique. Partial vestibulectomy was performed when necessary for adequate glottic exposure. Resections were performed with macroscopically clean surgical margins; in case of doubt, histological examination was performed using frozen section during surgery. The margins of the main specimen were marked with sutures and sent for definitive analysis by an experienced surgical pathologist.

### Data collection

We retrospectively collected data such as age, gender, smoking history, alcohol drinking history, tumor localization, T stage, cordectomy type, anterior commissure classification, CT findings, recurrence status and date, salvage treatment, and last examination date. Tumors were classified as AC1 to AC3 according to the ACI-specific classification proposed by Rucci et al.: “AC1, involves only one side of the midline; AC2, involvement of the anterior commissure subsite that crosses the midline on only part of the longitudinal extension of this subsite; AC3, involvement of the whole anterior commissure subsite on both sides across the midline [6]. Postoperatively, all patients were routinely evaluated every 4–6 weeks in the first year, every 2–3 months in the second and third years, and every 3–6 months in the fourth and fifth years. All follow-up data were accessed from patient database.

**Fig. 1:**
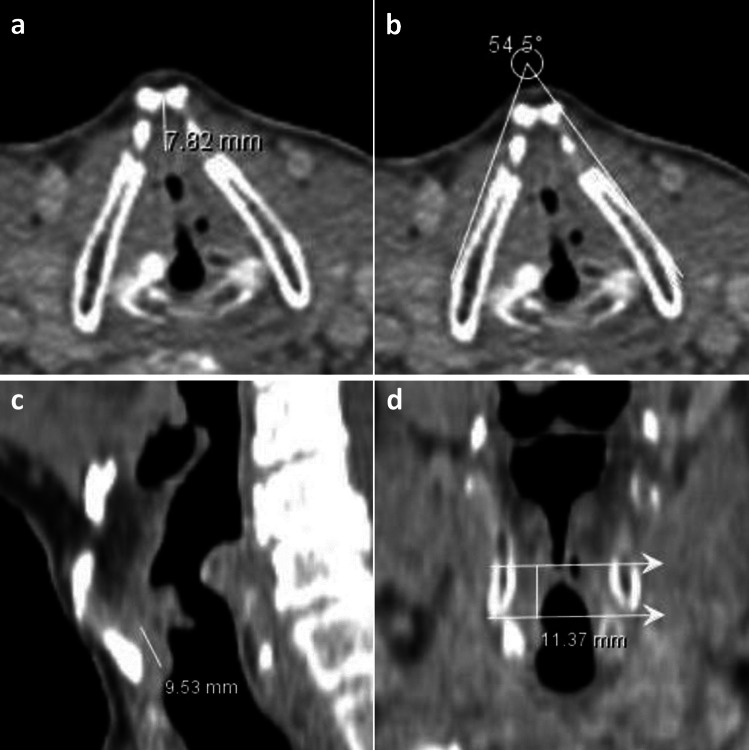
The computed tomography sections supplied depict representative instances of measurements for three parameters associated with the anterior commissure, which are utilized in the radiological assessment of patients involved in the study. These are listed as follows; **a** Measurement of the thickness of the anterior commissure in the section passing through the anterior commissure in the axial plane, **b** Measurement of the angle between the two laminae of the thyroid cartilage in the section passing through the anterior commissure in the axial plane, **c** Measurement of the vertical length of the tumor in the section passing through the anterior commissure in the sagittal plane, **d** Measurement in the coronal plane of glottic distance (vertical extension ratio was obtained by dividing the measurement in Fig. 1c to the measurement in Fig. 1d)

Radiological examination was conducted on three parameters (vertical extension ratio, anterior commissure thickness and thyroid cartilage interlaminar angle) associated with the AC. Images were obtained on a 64-detector CT device (Aquilion Toshiba) and Iohexal was used as contrast material (Omnipaque). Radiological evaluations were performed by two radiologists (4 years and 13 years of experience) unaware of the clinical and laryngoscopic findings. Evaluations were made on 2–3 mm thick axial, coronal and sagittal sections taken from the base of the tongue to the trachea, and a common decision was reached for each case. The glottic distance was measured in the coronal section soft tissue window in all patients. The vertical length of the tumor was measured in the sagittal section passing through the anterior commissure. This measurement was proportioned to the glottic distance measurement. In all patients, anterior commissure thickness and thyroid cartilage interlaminar angle were measured from the glottic distance in axial images (Fig. 1).

### Statistical analysis

Categorical variables were expressed as numbers and percentages, whereas continuous variables were summarized as mean and standard deviation and as median and minimum–maximum where appropriate. For comparison of continuous variables between two groups, the Student's t-test or Mann–Whitney *U* test was used depending on whether the statistical hypotheses were fulfilled or not. As main endpoints, the local control with laser alone (LCL) (includes all patients who underwent one or repeated laser surgery interventions and patients with recurrent disease treated with repeat laser surgery), recurrence free survival (RFS), and laryngeal preservation rate (LP) were assessed measuring the time from surgical resection to local recurrence for which non-laser treatments are applied, loco-regional/distant metastasis, and laryngectomy, respectively. Overall survival (OS) was defined measuring the time from surgical resection to death of any cause, and disease-specific survival (DSS) was defined by measuring the time from surgical resection to death due to laryngeal carcinoma. For univariate analysis OS, RFS, and LCL survival was calculated by Kaplan–Meier method and log rank test was performed. Cox regression analysis was performed to determine significant predictors of OS, RFS and LCL. In univariate analysis, variables significant at the *p* < 0.25 level were entered in multiple Cox regression (Backward Wald procedure) analysis. All analyses were performed using IBM SPSS Statistics Version 20.0 statistical software package. The statistical level of significance for all tests was considered to be 0.05.

## Results

**Fig. 2:**
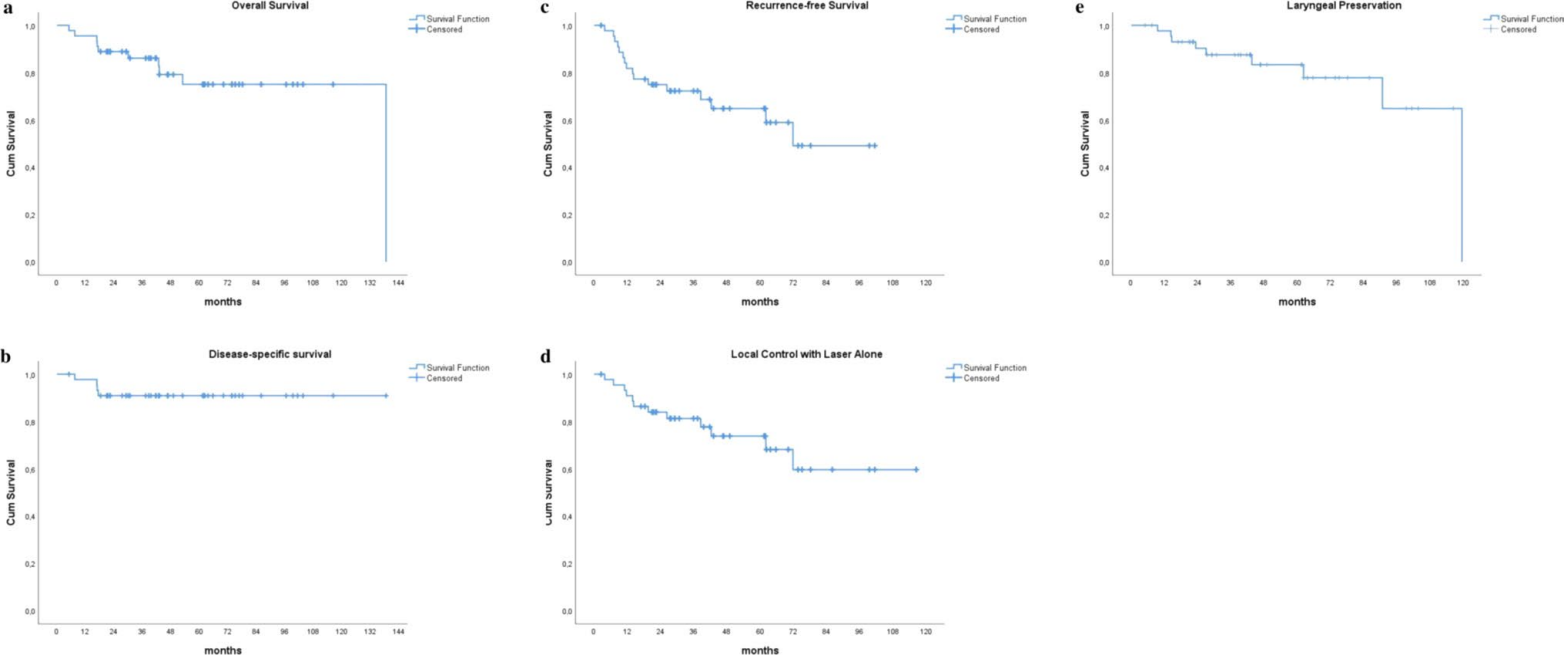
Kaplan–Meier curves for the study population. **a** Overall survival curve **b** Disease-specific survival curve **c** Recurrence-free survival curve **d** Local control with laser alone survival curve **e** Laryngeal preservation survival curve. Abbreviation: Cum Survival, Cumulative Survival

An evaluation was conducted on 89 consecutive patients diagnosed with early-stage glottic SCC involving the AC and treated with TLM between November 2011 and August 2021. A total of 28 patients with T2 stage tumors, 11 patients with positive surgical margins, and 5 patients with concomitant malignancy or who required open surgery during the same session or whose preoperative imaging and follow-up data were unavailable were excluded from the study. A total of 45 primary pT1N0M0 glottic carcinoma (based on the Eighth Edition of the American Joint Committee on Cancer TNM classification system) patients [41 (91.1%) males; mean ages 67.5 ± 9.3 years] with AC involvement (histopathologically confirmed SCC diagnosis), who were treated with CO2 TLM with negative surgical margins, were included in the study. Demographic, clinical and radiological characteristics of patients are presented in Table 1. Ten patients (22.2%) included in the study died. Local recurrence was detected in 16 patients (35%). As salvage treatment, four of these patients underwent repeated laser surgery, three patients underwent RT, three patients underwent partial laryngectomy and, six patients underwent total laryngectomy. In our series, mean OS, DSS, RFS and LCL were 111.5, 127.4, 66.9 and 84.7 months, respectively. 3- and 5-year OS probabilities were 86.1% and 75.1%, respectively. DSS probability was 90.9%, RFS were 72.3% and 64.8%, LCL were 81.3% and 73.8%, and LP were 87.6% and 83.4%, respectively. Survival curves are shown in Fig. 2.

**Table 1:** Demographic and clinic characteristics of study population

	*n* = 45
Gender, *n*(%)	
Male	41 (91.1)
Female	4 (8.9)
Age(year)	67.5 ± 9.3 68.0 (47.0–83.0)
Age	
< 75 years	34 (75.6)
≥ 75 years	11 (24.4)
Smoke, *n* (%)	43 (95.6)
Smoke	42.3 ± 23.7 35.0 (6.0–120.0)
Alcohol, *n* (%)	17 (37.8)
T stage	
T1a	25 (55.6)
T1b	20 (44.4)
AC Classification, *n* (%)	
AC1	18 (40.0)
AC2	14 (31.1)
AC3	13 (28.9)
Cordectomy type, *n* (%)	
Type 5a	27 (60.0)
Type 6	18 (40.0)
Thyroid cartilage interlaminar angle	71.2 ± 15.9 70.0 (48.0–115.0)
Vertical extension rate	0.16 ± 0.28 0.0 (0.0–1.0)
AC thickness(mm)	6.3 ± 2.2 6.0 (3.0–12.0)

Data were expressed as mean ± standard deviation, median(min–max)

*AC* Anterior commissure

### Clinical variables

**Fig. 3:**
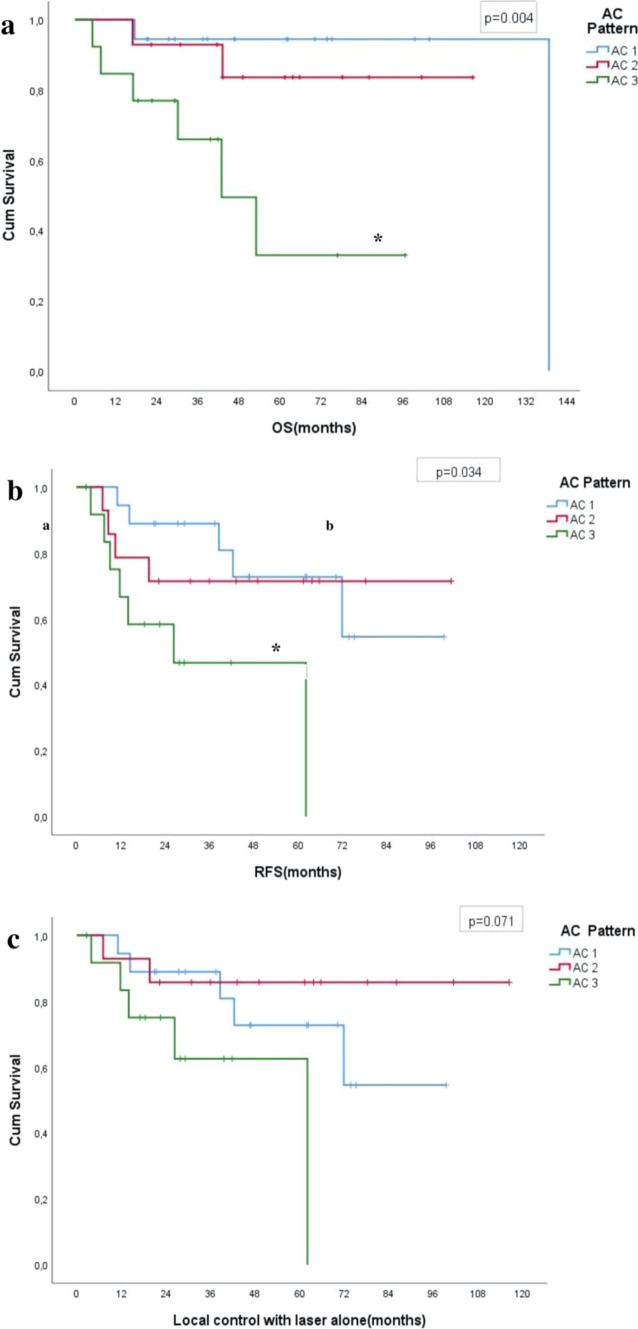
**a** Analysis of the overall survival of T1 tumors with respect to anterior commissure involvement pattern. *p = 0.004 compared with AC1 and AC2 by AC3. **b** Analysis of the recurrence-free survival of T1 tumors with respect to anterior commissure involvement pattern. *p = 0.034 compared with AC1 and AC2 by AC3. **c** Analysis of the local control with laser alone survival of T1 tumors with respect to anterior commissure involvement pattern. *AC* anterior commissure

Table 2 shows the results of survival analyses according to clinical factors. OS and RFS were higher in patients with early stages of AC pattern than in patients with advanced stage (*p = *0.004, *p = *0.034, respectively). Although median LCL were shorter in cases with AC3 compared with other AC patterns, differences were not statistically significant (*p = *0.071) (Fig. 3).

**Table 2:** Results of survival analyses according to clinical factors

	Total N/N of events	OS (months) mean/median	5-year OS	Total N/N of events	RFS (months) mean/median	5-year RFS	Total N/N of events	LCL (months) mean/median	5-year LCL
*T stage*									
T1a	25/5	117.2/138.7	81.2%	25/8	69.0/71.9	72.7%	25/6	75.1/–	80.0%
T1b	20/5	90.6/–	69.0%	20/8	64.5/–	55.8%	20/6	84.4/–	66.6%
*AC classification*									
AC1	18/2	131.9/138.7	94.4%	18/5	75.2/-	72.7%	18/5	75.2/-	72.7%
AC2	14/2	102.5/-	83.6%	14/4	75.7/-	71.4%	14/2	101.7/-	85.7%
AC3	13/6	53.2/42.9	53.0%	13/7	35.9/26.3	46.7%	13/5	44.5/62.1	62.5%
*Cordectomy type*									
Type Va	27/5	86.5/-	78.6%	27/10	65.7/-	61.1%	27/8	71.7/-	67.7%
Type VI	18/5	108.8/138.7	70.7%	18/6	67.5/71.9	69.5%	18/4	91.2/-	81.4%
Total	45/10	111.5/138.7	75.1%	45/16	66.9/71.8	64.8%	45/12	84.7/-	73.8%

*OS* Overall survival, *RFS* Recurrence free survival, *LCL* Local control with laser alone, *AC* Anterior commissure

OS, RFS and LCL curves did not differ according to T stage and cordectomy type (*p = *0.578 and *p = *0.931 for OS, *p = *0.496 and *p = *0.754 for RFS and *p = *0.711 and *p = *0.421 for LCL, respectively).

A Cox proportional hazards model was used to evaluate the potential predictors for OS, RFS and LCL. According to the analysis, only age, among the clinical variables, was found to be associated with OS (HR = 4.2, 95%CI:1.1–16.3, *p = *0.038). Other clinical variables could not be included in the model (Table 3).

**Table 3:** Cox regression analysis

OS					RFS				LCL			
Variables	Univariate		Multiple		Univariate		Multiple		Univariate		Multiple	
	HR (95%CI)	*p*	HR (95%CI)	*p*	HR (95%CI)	*p*	HR (95%CI)	*p*	HR (95%CI)	*p*	HR (95%CI)	*p*
Age(> 75 years)	5.3 (1.4–20.2)	0.014	4.2 (1.1–16.3)	0.038	2.4 (0.8–7.2)	0.134			1.5 (0.4–5.7)	0.549		
Smoke (package/year)	1.02 (1.00–1.04)	0.022			1.01 (0.98–1.02)	0.671			1.0 (0.9–1.1)	0.125		
*T stage*												
T1a	Ref	0.581			Ref	0.498			Ref	0.711		
T1b	1.5 (0.4–5.4)				1.4 (0.5–3.8)	0.498			1.2 (0.4–3.8)			
*AC classification*												
AC1	Ref	–			Ref	–			Ref			
AC2	2.4 (0.2–26.9)	0.466			1.2 (0.3–4.6)	0.766			0.5 (0.1–2.4)	0.371		
AC3	12.3 (1.5–103.1)	0.021			3.9 (1.2–13.3)	0.024			2.7 (0.8–10.3)	0.129		
*Cordectomy type*												
Type Va	Ref	0.931			Ref	0.754			Ref	0.425		
Type VI	1.1 (0.3–3.9)				0.9 (0.3–2.3)	0.754			0.6 (0.2–2.1)			
Thyroid cartilage interlaminar angle	0.98 (0.94–1.02)	0.447			0.98 (0.96–1.02)	0.478			0.96 (0.91–0.99)	0.041	0.96 (0.91–0.99)	0.041
Vertical extension rate	10.7 (1.9–61.3)	0.008	8.9 (1.3–58.9)	0.023	11.1 (2.7–46.0)	0.001	11.1 (2.7–46.0)	0.001	4.6 (0.9–28.9)	0.067		
AC thickness	1.3 (0.9–1.7)	0.071			1.1 (0.9–1.4)	0.362			1.1 (0.9–1.5)	0.412		

*OS* Overall survival, *RFS* Recurrence free survival, *LCL* Local control with laser alone, *HR* Hazard ratio, *CI* Confidence interval, *AC* Anterior commissure

### Radiological variables

The Cox proportional hazards model utilized to evaluate potential radiological predictors of OS, RFS, and LCL is indicated in Table 3. Vertical extension ratio was found to be associated with OS and RFS, and thyroid cartilage interlaminar angle with LCL by multiple Cox regression analysis.

The median thyroid cartilage interlaminar angle was 71.0 (min: 48.0, max: 115.0) in the group without recurrence and 64.0 (min: 48.0, max: 95.0) in the group with recurrence. Although the median thyroid cartilage interlaminar angle was larger in the group without recurrence, no statistically significant difference was found between the recurrence groups (*p = *0.367). Thyroid cartilage interlaminar angle distribution in AC patterns according to recurrence groups is shown in Table 4. In all three AC patterns, there was no difference in the distribution of thyroid cartilage interlaminar angles between recurrence groups (*p = *0.775 for AC 1, p > 0.999 for AC 2 and *p = *0.138 for AC3).

**Table 4:** Thyroid cartilage interlaminar angle distribution in AC patterns according to recurrence groups

AC Pattern	Recurrence		*p*
	No	Yes	
AC1	13	5	0.775
	75.2 ± 17.8	71.6 ± 19.3	
	71.0(54.0–115.0)	81.0(48.0–92.0)	
AC2	10	4	> 0.999
	68.2 ± 14.3	69.8 ± 19.8	
	68.0(48.0–97.0)	65.5(53.0–95.0)	
AC3	6	7	0.138
	76.0 ± 12.6	64.9 ± 15.4	
	77.5(59.0–90.0)	58.0(48.0–86.0)	

Data was expressed as *n*, mean ± ss, median(min–max)

*AC* Anterior commissure

## Discussion

The differential behavior of tumors involving the AC, in comparison to other glottic cancers, has been widely acknowledged, thus warranting specific consideration in the selection of treatment approaches. The impact of AC involvement on oncological outcomes is still controversial. While several authors argue that this condition lacks significance [7–9], others attribute it to the occurrence of local recurrence and a negative prognosis [2, 6, 10, 11]. Several potential explanations have been put out by proponents of the viewpoint that the involvement of the AC is associated with worse oncological outcomes. These are that T1 tumors have been observed to advance to T3 and T4 tumors by infiltrating the cartilage via perichondrium dehiscence at Broyles's ligament and the incidence of positive margins is notably elevated owing to the challenges associated with achieving comprehensive endoscopic visualization of this particular area [12].

The study conducted by Hoffmann et al., which is one of the largest series in the literature, including only cases with AC involvement treated with TLM, the 5-year OS, DSS, RFS, and LCL rates were found to be 79.2%, 91.5%, 61.7%, and 74.4%, respectively [2]. In the present investigation, despite the differences in patient groups, the rates of 5-year OS, DSS, RFS, and LCL were determined to be 75.1%, 90.9% 64.8%, and 73.8%, respectively, which aligns with the findings reported in the aforementioned study. Based on the findings in these studies and in comparison to existing international literature, it can be inferred that the involvement of the AC is associated with a decrease in survival rates. However, taking into account the tumor characteristics in patient series described in the literature, it seems likely that other factors, in addition to AC involvement, could also contribute to the decreased survival, including tumor stage, involvement of the pre-epiglottic and paraglottic regions, surgical margin positivity, etc. In the present investigation, some groups were excluded, resulting in the formation of a study cohort that exhibited homogeneity. This cohort was characterized by the presence of isolated T1 stage, negative surgical margins, absence of pre-epiglottic and paraglottic area involvement, and histopathologically confirmed AC involvement. Our goal was to identify AC-specific clinical and radiological tumor features in patients with T1 stage AC involvement and to investigate the impact of these features on tumor recurrence and survival.

The efficacy of the TNM classification in accurately predicting the likelihood of recurrence and unfavorable prognosis in instances of AC involvement remains a topic of contention. Certain specialists have posited that the TNM classification may not be appropriate as a prognostic indicator for the AC, and have proposed the development of a distinct classification system specifically tailored for this anatomical location. In parallel, Alkan et al. observed no discernible disparity in terms of recurrence rate, RFS or OS when comparing T1a and T1b tumors within the patient series they examined [12]. Our investigation yielded a comparable outcome. There was no significant difference in recurrence rate, OS, RFS and LCL rates between T1a and T1b tumors. Rucci et al. with similar thoughts, they proposed a specific classification for the AC and demonstrated that RFS decreased significantly as the AC score increased in their series of 33 patients [6]. In Carta et al.'s patient series, AC1 and AC2 involvement did not have a statistically significant effect on the patient's prognosis, but 5-year RFS was lower (74.1%, *p = *0.0446) in nine AC3 patients [13]. In the present study, similar to this cohort of patients, the impact of AC1 and AC2 participation on patient survival did not exhibit a statistically significant influence. However, it was shown that AC3 involvement resulted in a substantial reduction in both OS and RFS (*p = *0.004 and 0.034, respectively). Whether the tumor is bilateral in the shape of a horseshoe tumor or unilateral, we believe there is no difference in the prognosis for T1 glottic tumors with AC involvement. However, cranio-caudal involvement of the AC appears to be associated with a high relapse rate and poor survival. Furthermore, within the scope of our investigation, we utilized CT to measure the vertical distance in the AC and afterwards proportioned it to the measurement of the glottic distance. In the context of multivariate analysis, it was observed that an elevated ratio of vertical extension to glottic distance had a substantial negative impact on OS and RFS (HR = 8.9, 95%CI 1.3–58.9, *p = *0.023 and HR = 11.1, 95%CI 2.7–46.0, *p = *0.001, respectively). The ratio in question is considered to be a valuable measure for predicting survival. Indeed, while the existence of the aforementioned unfavorable prognostic indicators does not serve as an absolute prohibition for TLM, it is advisable for the surgeon to adopt a more extensive resection approach and closely monitor the patient through repeated direct laryngoscopy and biopsy in the presence of these factors.

One point that should be noted regarding AC involvement is to evaluate whether the tumor originates from the AC or originates from another region of the vocal cord and involves the AC. Although current endoscopic and radiographic procedures provide information regarding the tumor's location and spread pattern, it may not always be possible to make this distinction clearly. Nevertheless, the fact that the tumor volume is predominantly in the vocal cord or AC may hint at the tumor's origin. Additionally, horseshoe tumors typically begin in a vocal cord, involve the AC, and move horizontally to the opposing vocal cord. It is thought that these tumors have less potential to invade the Broyles ligament and have a better prognosis than vertically extending tumors [12]. Tumors originating from the AC have a significant likelihood of impacting all AC subsites. Secondarily, these tumors also show a vertical progression pattern. Although we were unable to pinpoint the origin of all tumors in our investigation, tumors with vertical extension were primarily from the AC. T1a tumors, which involved just one vocal cord and the AC, and T1b tumors, which involved the AC only horizontally in a horseshoe form, were tumors that mostly originated from the vocal cord and involved the AC. Our investigation observed that tumors originating from the AC were predominantly vertically extending tumors and AC3 type involvement according to Rucci’s classification [6]. In terms of survival and recurrence rate, these tumors tended to have a poorer outcome than those originated from the vocal cord [2, 7, 13]. However, due to the challenges in precisely identifying the region of the tumor’s origin, it is not possible to draw a definitive conclusion. In the study examining the effect of radiological tumor thickness on local recurrence, evidence was found that tumor thickness measured under CT guidance is a predictive factor for tumor recurrence [14]. In the current investigation, we sought to identify the association between local recurrence and the anterior commissure thickness assessed under CT guidance. No significant correlation was observed between the thickness of the AC and the recurrence of the disease or the OS rate. In addition to the fact that the thickness increase in the AC is dependent on the tumor tissue, it is believed that inflammatory and infectious incidents that are secondary to the tumor can contribute to the situation. Furthermore, it can be predicted that tumors with a relatively benign course and an exophytic character with a low depth of invasion will increase in thickness in this region.

**Fig. 4:**
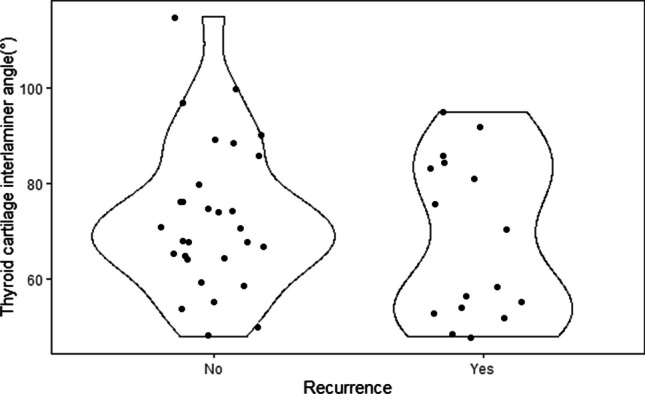
Display of thyroid cartilage interlaminar angle values respect to recurrence status in the violin plot

One of the potential explanations for the association between involvement of the AC and a negative prognosis is the challenge in achieving comprehensive exposure of this specific region. Achieving complete visualization might provide challenges, particularly in instances involving narrow-angle and V-shaped thyroid cartilage [15]. The presence of a thyroid cartilage with a narrow angle poses challenges not only in terms of exposure, but also in accessing the region where the Broyles ligament attaches to the thyroid cartilage. The treatment of laser surgery for the AC involves the dissection of the inner side of the cartilage that connects to the Broyles ligament in the subperichondrial plane, followed by the removal of the dense fibroelastic tissue in this region. In the context of our clinical practice, it is anticipated that the narrow angle of the thyroid cartilage poses challenges during dissection procedures. Specifically, difficulties may arise when dealing with tumors that extend in the cranio-caudal direction, as these have a heightened propensity for invading this particular anatomical region. The existing literature lacks an examination of the thyroid interlaminar angle. In the conducted multivariate analysis, although the narrowing of the angle in question did not have a statistically significant effect on OS and RFS, it was determined that it significantly reduced the LCL (HR = 0.96, 95%CI 0.91–0.99, *p = *0.041). Additionally, particularly in cases involving AC3 involvement it was seen that the median value of the group experiencing recurrence exhibited a narrower angle compared to those without recurrence (58 vs. 77.5 degrees). However, this difference did not yield statistically significant results. Furthermore, a definitive cutoff for this angle that accurately predicts the likelihood of recurrence could not be determined. The noted situation can be attributed to the insufficiency of the sample size. In instances when the angle measures 60 degrees or less, the observed recurrence rate is 57.1% (8 out of 14 cases) (Fig. 4). In light of these observations, it can be possible to attain relevant results by studying larger case series.

While there is ongoing debate on the impact of AC involvement on oncological outcomes, the prevailing consensus suggests that it is associated with a negative prognosis. The selection of the appropriate procedure also remains a subject of ongoing discussion. Previous studies have reported poor outcomes in terms of recurrence rates for AC involved tumor that were treated with TLM [2, 10, 11, 16–18]. On the other hand, AC involvement has not been found to be a significant indicator for oncological outcomes in several TLM investigations [8, 12, 19]. For radiotherapy, although the negative role of AC involvement has been defined in some studies [20, 21], there are also studies reporting that AC involvement has no significant effect on oncological outcomes [12, 22, 23]. Some studies have reported that RT does not show a statistically significant difference in terms of OS and RFS when compared to surgical treatment alternatives [24–26]. However, in the review by Hendriksma et al., the 5-year local control rates of T1 stage patients with AC involvement receiving TLM and RT treatments were found to be 70.1 and 82.2, respectively [27]. They also observed that the majority of the RT studies did not indicate that AC involvement had a significant impact on oncological outcomes. Open surgery, another treatment approach, is an effective conservative strategy in terms of oncological outcomes in tumors involving AC, despite some disadvantages such as higher perioperative morbidity and the need for tracheotomy [28, 29]. Nevertheless, when examining research that compares open surgery with alternative treatment approaches, Li et al. with RT [30] and Jacobi et al. with TLM [31] revealed no significant difference in terms of oncological outcomes in their comparison. Additionally, even if there were better oncological outcomes, the question remains whether this would offset the more invasive approach. While the role of AC in the prognosis and treatment of early-stage glottic carcinoma remains a matter of debate, detailed classification of tumors involving AC appears necessary to further elucidate this role.

The oncological results we got utilizing TLM in our investigation, which employed the AC classification [6], were consistent with previous research findings. It was demonstrated that poor prognosis was associated with AC3 type involvement and elevated vertical extension ratio. Furthermore, we have acquired evidence suggesting that a narrow-angle thyroid cartilage may be linked to an unfavorable outcome due to its potential to impede exposure and resection procedures. Consequently, it is imperative to conduct an individualized assessment of each patient's therapy options in order to determine the most optimal course of treatment. TLM is a safe and reliable surgical technique with less morbidity that can be repeated in selected tumor recurrences, although AC involvement has a significant negative impact on outcomes. However, when taking into account the rate of recurrences in cases involving AC3 type, significant vertical spread, and narrow-angle thyroid cartilage, it may be appropriate to consider close follow-up or alternative therapeutic approaches, Nevertheless, as far as we are aware, there is a lack of research that utilize the aforementioned factors to assess alternative treatment methods. Further research is required to examine these factors in alternative treatment methods in order to ascertain the comparative advantages of different approaches. Our study had some limitations. Inherent in such retrospective studies is selection bias due to lack of randomization. Additionally, selection bias may have been amplified by the application of exclusion criteria. The number of patients included in the cohort was low due to the high number of exclusion criteria. In interpreting oncological results, it should be taken into account that although the number of patients in our study was similar to other studies, statistical power generally decreased after classification of subgroups. Another limitation is that due to the lack of patient groups receiving other treatment modalities in our study, comparisons could not be made between modalities. In addition, the histopathological features of the tumor were ignored in our study.

## Conclusion

In the decision to treat T1 stage glottic carcinomas involving the AC with TLM, it is of paramount importance to evaluate each patient individually and meticulously examine them endoscopically and radiologically. Although the AC involvement does have an unfavorable impact on outcomes, TLM is an efficient, quick, and minimally invasive treatment option for the majority of these patients. In this cohort, AC3-type involvement and elevated vertical extension ratio were the statistically poor prognostic indicators following TLM, although T classification was not. Additionally, we obtained clues that the presence of narrow-angle thyroid cartilage will cause difficulty in exposure and resection, leading to a negative prognosis in patients with AC involvement. It must be taken into account to keep in mind alternative modalities while making treatment decisions for these cases.

## Data Availability

The data supporting the findings of this study are available from the corresponding author upon reasonable request.
